# Acute total occlusion of the left main coronary artery treated with percutaneous intervention and simultaneous implantation of intra‐aortic balloon pump

**DOI:** 10.1002/ccr3.1227

**Published:** 2017-11-08

**Authors:** Vojko Kanic, Damijan Vokac, Samo Granda

**Affiliations:** ^1^ University Medical Center Maribor Maribor Slovenia

**Keywords:** ECG, intra‐aortic balloon pump, left main coronary artery occlusion, primary PCI, shock, ST‐elevation myocardial infarction

## Abstract

Electrocardiographic findings at first medical contact and direct transfer to the catheterization laboratory are important in acute total occlusion of the left main coronary artery. Simultaneous emergency angioplasty and intra‐aortic balloon pump implantation might be beneficial in overcoming the patient's most critical hemodynamic instability.

## Introduction

Acute total occlusion of the left main coronary artery (LMCA) is an uncommon clinical emergency. The presentation of patients with acute LMCA occlusion is usually catastrophic in comparison with occlusion of a more distal coronary bed as they usually present with sudden death or profound cardiogenic shock (CS) due to malignant arrhythmia or pump failure 1, 2. Most patients die without immediate treatment 3. The data on acute total occlusion of the LMCA with ST‐elevation myocardial infarction (STEMI) and CS are relatively limited.

We present the case of a 64‐year‐old man with acute total occlusion of the LMCA presenting as STEMI with CS who was treated with emergency percutaneous coronary intervention (PCI) with simultaneous implantation of an intra‐aortic balloon pump (IABP).

## Case Report

**Figure 1:**
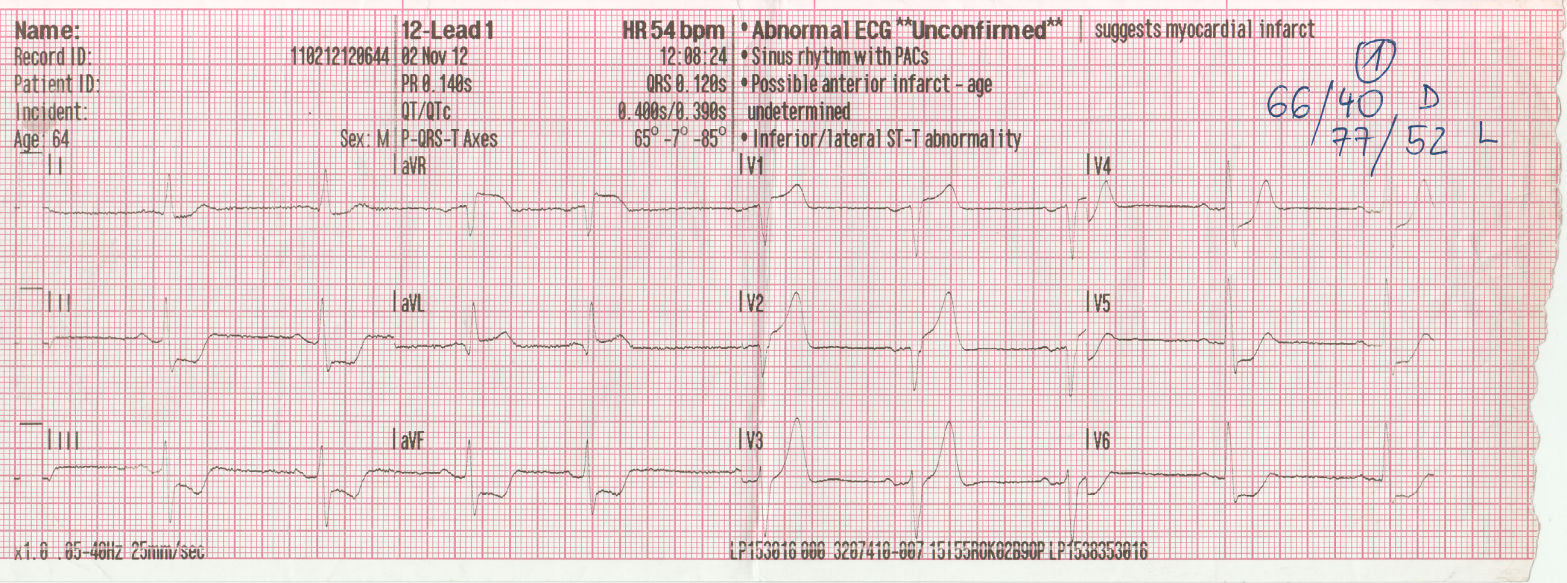
Electrocardiogram at first medical contact.

A 64‐year‐old male patient without previous medical history called the emergency physician because of chest pain lasting for 30 min. Hypotension (66/40 mmHg) and sinus bradycardia (52 beats/min) were present at first medical contact. The ECG showed ST‐segment elevation in leads aVR, aVL, and V1‐V2, as well as ST‐segment depression in the inferolateral leads, and LMCA pathology was suspected (Fig. 1).

Three hundred milligrams of aspirin intravenously was administered, and direct transport to the catheterization laboratory was arranged immediately. Heparin was not given because bivalirudin administration was anticipated in less than 30 min. CS, anticipated LMCA pathology, and the possible need for urgent surgery obviated the administration of P2Y12 receptor antagonist. Crystalloid infusions and 15 μg/kg/min of dopamine were given. Defibrillation was required because of ventricular fibrillation during transport.

**Figure 2:**
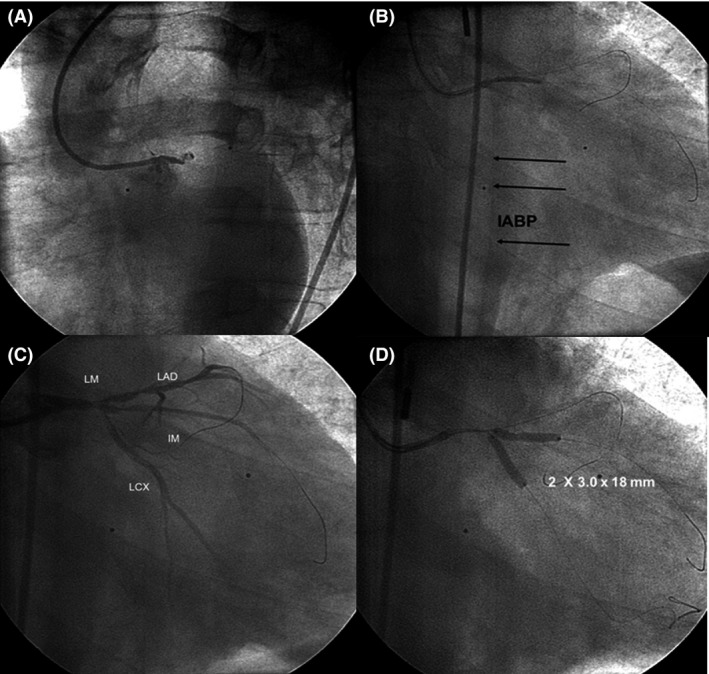
(A) occlusion of the LMCA; (B) IABP already functioning; (C) thrombotic trifurcation lesion in the LMCA; (D) LCX and IM stenting with the V‐technique. IABP, intra‐aortic balloon pump; IM, intermediate artery; LCX, circumflex artery; LMCA, left main coronary artery.

On admission, his blood pressure was 50 mmHg, pulse rate was 52 beats/min, and some pulmonary rales were present. Norepinephrine (30 μg/min), oxygen (6 L/min), and a bolus of bivalirudin (0.75 mg/kg) followed by an infusion (1.75 mg/kg/h) were administered. We surmised that pathology of the LMCA was present and started with a 7F extra backup 3–75 guiding catheter through the right femoral artery while the second operator simultaneously began to implant the IABP into the left groin. The first image of the left coronary artery revealed complete occlusion of the LMCA with TIMI 0 flow (Fig. 2A). We started with PCI of the LMCA without visualization of the right coronary artery (RCA).

After the first guide wire passed through the LMCA toward the left anterior descending artery (LAD) and a balloon angioplasty was performed with a 2‐ to 0‐mm balloon, no flow returned. The second wire went into the intermediate artery (IM). While performing balloon inflation with a 2‐ to 0‐mm balloon, the IABP was already functioning (Fig. 2B).

TIMI 2 flow revealed a large thrombus in the distal LMCA and a true trifurcation lesion (Medina 1,1,1,1). The third guide wire was introduced into the circumflex artery (CX) (Fig. 2C).

Two drug‐eluting stents (DES) (3–0 x 18 mm) were directly implanted in the ostial CX and ostial IM using the V‐technique (Fig. 2D).

**Figure 3:**
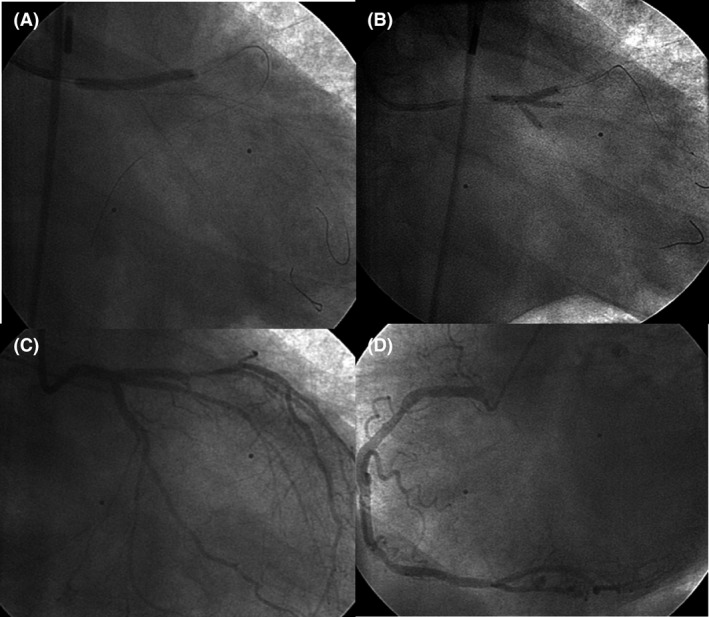
(A) stent in LMCA and LAD; (B) final kissing; (C) final result; (D) RCA. LMCA, left main coronary artery; LAD, left anterior descendant artery; RCA, right coronary artery.

A DES (3–5 x 33 mm) was directly inserted into the ostial LMCA toward the LAD to cover the whole LMCA and the proximal stenosis of the LAD, and postdilatation of the proximal LMCA with a 4‐ to 5‐mm noncompliant balloon was performed (Fig. 3A). Final kissing with three noncompliant balloons (Fig. 3B) achieved TIMI 3 flow in the LAD and the CX; however, TIMI 2 persisted in the IM (Fig. 3C). As soon as the procedure on the left coronary artery was completed, the RCA was revealed (Fig. 3D). There was almost no collateral flow to the left system.

Prasugrel (60 mg) was given approximately 1 h after the procedure. Despite the IABP and inotropic support, the blood pressure did not exceed 80 mmHg and intubation was required. Two days later, we were able to stop the IABP. The patient was extubated after 6 days. He was discharged after 17 days with an EF of 45% and in good clinical condition. He lives a normal life.

## Discussion

The optimal revascularization modality (CABG or PCI) for patients with acute total occlusion of the LMCA is still unknown 4. Emergency PCI of the LMCA with the deployment of DES in high‐risk patients gives results equivalent to that of CABG 4. Only scarce and inconsistent data have been reported with regard to primary PCI for STEMI due to acute LMCA occlusion 1.

The observed mortality rate of these patients is around 60% and around 80% in patients presenting with CS, although this might exceed 90% 1, 2. Early revascularization in patients with CS, either by surgery or PCI, increases the 1‐year survival compared to survival after aggressive initial medical stabilization 5. Patients with shorter pain to reperfusion time have a higher survival rate 3.

Emergency CABG in patients with STEMI complicated by CS may be effective but time‐consuming, and carries the risk of extensive and irreversible myocardial damage 4. PCI permits rapid reperfusion of the LMCA 4, 6. PCI seems to be a better choice of treatment than CABG for this special subset of patients with regard to the time to reperfusion. An organized network for primary PCI in STEMI patients may further encourage the percutaneous approach. At first medical contact, occlusion of the LMCA might be suspected from the electrocardiographic changes and clinical presentation 7, 8. ST‐elevation in lead aVR with anterior ST‐elevation or ST‐segment depression in leads V4–V6 on the electrocardiogram may indicate left main occlusion 9. The emergency physician should immediately alert the nearest seven of 24 hospitals about the patient's critical clinical condition and arrange direct transport to the catheterization laboratory. In our case, the emergency physician had recognized the possibility of LMCA pathology and alerted us in advance which was most probably lifesaving.

As most of the patients with acute occlusion of the LMCA develop a sudden, profound deterioration in hemodynamics, simultaneous efforts to maintain the systemic circulation and to achieve reperfusion of the occluded LMCA as soon as possible are essential for survival 2. Emergency reperfusion of the LMCA, under stabilizing measures such as the insertion of an IABP, is the primary goal in patients with acute LMCA occlusion 4. Despite its common use in clinical practice, there is conflicting evidence of the benefit of IABP in CS 10. The available data are not really representative of patients with acute total occlusion of the LMCA. In the Shock II trial, only around 9% of patients had LMCA pathology and there were no data on acute occlusion of the LMCA 11. In clinical practice, these patients usually undergo IABP implantation because left ventricular assist devices (LVAD) are normally not available 10. Simultaneous IABP implantation during PCI may overcome the most critical hemodynamic instability until the real cause (acute total occlusion of the LMCA) is resolved. Implantation of the IABP simultaneously with a PCI procedure seems to be a better choice than IABP implantation before (or after) PCI. We propose that two operators work simultaneously in such patients (one for PCI and the other for IABP implantation) whenever possible. Simultaneous insertion of an easily implantable LVAD may be the best logical choice for these high‐risk patients. However, there are no relevant data on this issue.

The presence of collateral blood flow to the left coronary artery or a dominant right coronary artery may help to preserve the left ventricular function once the event occurs 3. However, it is highly unlikely that the patient with acute total occlusion of the LMCA will survive in the long run despite a dominant RCA, without a quick restoration of the flow through the LMCA. Once the acute occlusion of the LMCA is observed at angiography in patients with CS, there is no need to visualize the RCA before the LMCA is repaired (if a percutaneous treatment is chosen). The RCA can be visualized after PCI of the LMCA.

## Conclusion

We conclude that the following three important steps enabled the successful treatment of our patient:

1. The emergency physician recognized the LMCA involvement, notified the catheterization laboratory at first medical contact, and arranged direct transport to the catheterization laboratory.
2. Simultaneous implantation of IABP during PCI helped to maintain the patient's hemodynamic status during the critical ischemic time.
3. PCI of the LMCA without wasting time to show the RCA anatomy shortened the ischemic time.

We propose these measures whenever acute LMCA occlusion is suspected and LVAD is not available. This should shorten the time from first medical contact to revascularization and potentially improve the hemodynamic instability. However, further investigation is needed to prove our concept.

The pain to first medical contact time will not be influenced by these measures. This time still mostly depends on the patient's knowledge and quick reaction at the onset of chest pain. The possible role of CABG (when needed) in addition to PCI in this special subset of patients still needs to be determined. Despite optimal treatment, there will still be a high mortality in these high‐risk patients.

## Authorship

VK: wrote the manuscript and revised intellectual and technical content of the manuscript. DV, VK, and SG: reviewed the manuscript.

## Conflict of Interest

None declared.
